# Supplementary material for: Are carbon emissions trading and green financial instruments synergistic? -Comprehensive quantitative research based on content analysis
Source: PLoS One. 2024 Mar 7;19(3):e0298601. doi: 10.1371/journal.pone.0298601 (PMC10919601; doi:10.1371/journal.pone.0298601)
Supplement: S1 Data — (ZIP) [file pone.0298601.s001.zip › Data/地级市Z_F工作报告.zip!/╦─┤¿/┼╩╓a╗¿╩╨/└┤╫╘╣1⁄2╓┌║┼í╛┬φ┐╦╩2╛▌═°í┐_2018.docx]

各位代表：

　　我代表市人民政府，向大会报告工作，请予审查，并请市政协委员提出意见。

2017年工作回顾

　　2017年是本届政府的开局之年。一年来，在省委省政府和市委的坚强领导下，在市人大和市政协的监督支持下，市政府认真学习贯彻党的十九大精神，坚持以习近平新时代中国特色社会主义思想为统揽，全面落实省第十一次党代会、市第十次党代会和市委十届二次全会部署，坚持稳中求进工作总基调，统筹推进稳增长、促改革、调结构、惠民生、防风险各项工作，全力做好“钒钛、阳光”两篇文章，推动经济社会各项事业取得新的成绩。全年实现地区生产总值1144.25亿元，增长7.4%；全社会固定资产投资745.28亿元，增长7%；社会消费品零售总额352.41亿元，增长11.2%；地方一般公共预算收入60.59亿元，增长6.8%；城镇、农村居民人均可支配收入分别为35620元、15336元，增长8.4%、9.1%；城镇登记失业率4.01%。

　　一、经济增长稳中有进

　　工业经济企稳回升。完成规上工业总产值1797.09亿元，增长19.4%；规上工业增加值增长7.5%；工业投资238.65亿元、技改投资257.35亿元，分别增长2.2%、8.1%。投资20亿元的工业园区第一批基础设施PPP项目开工，为优质产业项目入驻奠定了基础。攀钢产品结构优化调整改造等重大项目竣工投产，高炉渣提钛产业化示范项目低温氯化工程、银江水电站岸上工程等项目开工建设。攀钢等大企业扭亏为盈，四大工业企业完成工业总产值602.7亿元，增长19.6%，124户停产限产规上企业恢复正常生产。全市升规企业35户，工业行业增长面达87%，主营业务收入增长23.8%，创近年来新高。

　　现代服务业发展加快。完成服务业增加值362.07亿元，增长8%。房地产开发投资59.96亿元、销售商品房175.58万平方米，实现“双增长”。限上商贸企业新增20户，电商线上交易额增长30%。82户企业在天府联合股权交易中心挂牌，总规模100亿元的钒钛资源开发投资基金、50亿元的康养产业投资基金组建运行，成为全省第一批促进科技与金融结合特色试点城市。超额完成旅游厕所3年行动计划，实现旅游收入279亿元，增长15%。

　　现代农业提质增效。积极推进农业供给侧结构性改革，完成农林牧渔业总产值65.88亿元，增长4.5%。中国热科院攀枝花实验基地开工建设，新创建热作标准化示范园4个，完成马鞍山、梅子箐水库主体工程。新增“三品一标”农产品10个。攀枝花芒果入选首批中国特色农产品优势区、2017年度中国百强特色农产品区域公用品牌，远销俄罗斯、新加坡、香港等国家和地区，出口突破2万吨，增长33.2%。

　　“康养+”产业项目化、具体化。在全国率先发布康养产业地方标准。攀西兰花谷等33个农康产业基地景区加快建设，成为国家康复辅助器具产业综合创新试点地区，攀枝花国际康养学院建成投用，成功承办全球华人羽毛球锦标赛等一批高水平运动赛事，攀枝花康养旅游综合体获评全国“2017年度民生示范工程”。

　　抓项目、抓政策、抓资金成效明显。扎实推进“项目年”工作，推行“五个一”项目工作机制，出台加强和改进投资促进工作的23条措施，开展专业化招商，新签约国电龙源电力等项目290个，实际到位资金663.19亿元，增长16.7%。争取攀西试验区“五大领域”先行先试政策，国土资源部支持省上出台了促进攀枝花矿业城市转型发展12条措施。争取到上级资金178.99亿元，增长10.1%。

　　经济增长保持了稳中有进、稳中向好的良好态势，三次产业结构由3.4：69.0：27.6调整为3.2：65.2：31.6，科技对经济增长的贡献率提高0.5个百分点、达55.5%，高新技术产业产值占工业总产值比重提高0.5个百分点、达25.5%，产业结构调整和转型发展巩固了持续向好的势头。

　　二、深化创新改革取得新进展

　　供给侧结构性改革成效显著。深入推进“三去一降一补”，退出低效煤炭产能87万吨，淘汰落后钢铁产能25万吨。深化银政企合作，红名单企业融资成本下降0.8个百分点。为企业争取铁路运费优惠2.7亿元，地方售电公司实质运营，直购电用电门槛标准下降50%。规上工业企业资产负债率下降7个百分点。攻克特殊钢高效洁净低成本生产工艺等关键技术。成功创建四川省质量强市示范城市。

　　重要领域和关键环节改革任务落地落实。深化“放管服”改革，精简行政权力事项815项，实施容缺审批服务，工业、房地产项目审批时限分别压缩到33个、25个工作日。深入推进商事制度改革，新登记市场主体1.26万户、增长13.7%。深化国资国企改革，“三供一业”分离移交有序推进。农业农村改革加快推进。出台《攀枝花人才新政七条》。

　　全面创新改革试验系统推进。钒钛“五个一工程”成效明显，建成国家级钒钛检测重点实验室，制定钒钛产业国家标准4项、行业标准20项，国、省级创新创业平台达26家。军民融合取得新进展，攀钢产品进入军工领域。

　　三、扩大开放合作取得新成绩

　　对外大通道建设取得新进展。新增1个至成都航班，机场迁建、通用机场前期工作同步开展。成昆铁路扩能改造工程加快推进，大（大理）攀昭（昭通）高铁争取工作取得阶段性进展。攀大（大理）高速攀枝花段主体工程进度过半，宜（宜宾）攀高速开工建设，攀盐（凉山盐源）高速启动工程可行性研究。普通国省道路况持续改善。

　　区域合作取得新成效。与成都市在交通、康养、金融、干部人才等十个方面开展深度合作。与川滇黔峰会成员市（州）的合作进一步加强，联合争取大（大理）攀昭（昭通）铁路按照高铁标准建设。与北京市西城区、海淀区合作持续深化，在北京、上海、香港、澳门等地举办芒果品牌发布推介活动。开启攀枝花与苏锡常都市圈区域合作的新篇章。成立康养产业城市联盟的倡议得到18个城市积极响应。

　　对外开放迈出新步伐。成功举办攀枝花钒钛资源综合利用院士行暨钒钛（国际）论坛、第三届中国康养产业发展论坛等活动，全年招引省外项目77个。主动加强与“一带一路”沿线国家和地区的交流互动，十九冶中标海外项目59项、总金额2.2亿美元。全市外贸进出口总额26.2亿元，增长89.3%，增幅居全省第二位。

　　四、保障和改善民生取得新成效

　　下足“绣花”功夫完成年度脱贫目标。完成677户2834人易地扶贫搬迁，721户农村危房改造，35个贫困村退出，8881名贫困人口脱贫的年度任务。扎实推进“六大扶贫工程”，建立四项扶贫基金1.29亿元，发放教育卫生和产业扶持基金3230万元，撬动扶贫小额贷款2.06亿元，惠及贫困人口4.17万人次。

　　城乡建设管理水平不断提升。完成城市总规、城市轨道交通线网规划等修编工作。总投资75亿元的东区后山道路等7个市政基础设施PPP项目开工建设，花城新区建设加快，花街花道花园花海第一期建设初步完成，城市承载能力进一步提升。推进城市执法体制改革，城市精细化管理水平持续提高。建成幸福美丽新村40个、省级“四好村”20个、市级“四好村”100个。新（改）建农村公路320公里。

　　民生实事推进有力。全面完成十大民生工程和十件民生实事，民生支出占一般公共预算支出超过65%。“9·19”灾后恢复重建基本完成。新建（改扩建）一批公办幼儿园、公园绿地、停车场、农贸市场。棚户区改造新开工2.2万套。成为全国老旧小区改造试点城市、中央财政支持的居家和社区养老服务改革试点地区。

　　社会事业全面进步。新增城镇就业1.8万人，就业促进、社会救济等保障体系更加完善。新增普惠性民办幼儿园17所，公办和普惠性幼儿园学位比例达80%，攀枝花成都外国语学校建成投用，普通高考本科上线人数实现九连升，建成国家级技能大师工作室3个，我市选手第二次获得世界技能大赛焊接项目金牌。扎实推进公共文化服务体系建设，对农广播媒体融合传播指数进入全国百强。完成城乡居民医保整合，10家医院接入异地就医即时结算国家平台，成为全国首个通过国家省市三级联动测试的地级市；引入北京301医院、四川大学华西医院等优质资源；公立医院取消药品加成累计让利3.2亿元，次均住院费用降幅居全省前列。全面二孩政策平稳有序实施。

　　五、社会治理水平实现新提升

　　完善社情民意收集回应机制。召开“我与市长面对面”“县（区）长面对面”市民座谈会，常态化听取市民和企业对政府工作的意见建议。全年办理12345市民热线和“市长信箱”反映事项18682件，办结率100%、抽查满意率100%。

　　社会大局保持稳定。深入开展平安攀枝花建设，刑事、治安案件分别下降40.7%、32.6%，平安建设群众满意度测评居全省第二位，米易县获“全国平安建设先进县”称号，圆满完成党的十九大等特殊敏感时期安保维稳任务。建立完善信访约谈、联合接访、顶岗跟班、市级领导包案等机制，市政府常务会每月专题研究信访工作，推动信访维稳形势进一步好转，信访批次、人次分别下降16.3%、48.3%，案件化解率、群众满意率分别上升34.2%、12.7%，30件省交办案件全部办结。中央环保督察期间，我市信访总量较少。

　　安全保障扎实有力。安全生产事故发生率、死亡率分别下降45.33%、24.07%。治理地质灾害隐患点19个，整改防汛隐患622个，集中开展极端气象条件下防汛应急综合演练2次，成功应对“6·28”暴雨灾害天气。食品药品安全保障水平进一步提升。

　　生态环境持续改善。集中开展“四大专项行动”，中央环保督察交办的123件信访件全部办结，省环保督察反馈的237个问题已整改210个，省挂牌督办的89户选矿企业全部关停取缔。全面实行河长制。入选四川省山水林田湖生态保护修复示范试点。强力推进污染防治“三大战役”，空气质量全面达标、优良率98.4%，城市污水处理率93.3%，地表水和饮用水水源地水质达标率100%。成功创建国家森林城市、国家园林城市。

　　六、政府自身建设切实加强

　　法治政府建设深入推进。坚持民主集中制，健全完善“三重一大”事项决策机制，严格落实常务会学法制度，行政执法三项制度全面实施，首部实体地方性法规《攀枝花市城市绿化条例》经市人大常委会审议通过。主动接受人大法律监督、工作监督，自觉接受政协民主监督和社会各界监督，办结人大代表建议94件、政协提案222件，办复率100%、满意率100%。

　　专业能力不断提升。扎实开展“两转一提”，全面提升干部队伍专业能力。实施对标管理，出台《关于全面开展对标管理的实施意见》，学习成都、苏锡常等先进地区的发展理念和工作机制。开展项目竞进拉练，县（区）和部门项目意识增强，比学赶超的竞争态势初步形成。开展双向交叉测评，强化跟踪督办，政务服务效能不断提升。

　　党风廉政建设不断加强。严格落实全面从严治党要求，常态化制度化开展“两学一做”学习教育。深入贯彻中央八项规定精神，坚决反对“四风”。认真履行“一岗双责”，扎实推进政府系统惩治和预防腐败体系建设。强化行政监察和审计监督，查办违纪违法案件446件448人，党纪政纪处分429人。党风廉政建设社会测评居全省第二位，党风廉政建设持续向好。

　　此外，国家安全、国防动员、双拥、消防、人防、对台和外事侨务等工作切实加强，民族宗教、妇女儿童、青少年、老龄、残疾人、慈善等工作取得新进步，统计、供销、粮食、档案、邮政、地方志、机关事务管理、关心下一代等工作取得新进展。

　　各位代表，过去一年，全市干部群众砥砺奋进，取得了显著成绩，全面完成了市十届人大一次会议确定的目标任务。这些成绩的取得，是省委省政府和市委正确领导的结果，是市人大、市政协和社会各界监督支持的结果。在此，我代表市人民政府，向在各个领域和岗位上辛勤劳动、作出贡献的全市人民；向给予我们监督和支持的人大代表、政协委员，各民主党派、工商联、知联会、新阶联和无党派人士，各人民团体、社会各界人士；向鼎力支持我们工作的驻攀部门和企业，驻攀解放军指战员、武警官兵、政法干警和民兵预备役人员；向各位老领导、老同志以及所有关心支持攀枝花发展的同志们、朋友们，表示衷心的感谢！

　　我们也清醒地看到，全市人民日益增长的美好生活需要和不平衡不充分的发展之间的矛盾依然突出：对外高效便捷通道不畅，物流成本较高仍是制约发展的重要瓶颈；经济总量较小与产业转型升级的矛盾并存，转型发展仍处于爬坡上坎的关键时期；工业园区基础设施配套不完善，重大产业化项目落地难，项目支撑不足；城市基础设施欠账较多，优质公共服务还不够均衡；民生保障水平和质量有待进一步提升；一些政府工作人员的观念、作风、专业能力、专业精神还不适应新时代发展的要求。这些问题，我们一定努力加快解决，不辜负全市人民的期盼！

　　2018年工作安排

　　2018年，是贯彻党的十九大精神的开局之年，是改革开放40周年，是决胜在全省高水平率先全面建成小康社会的冲刺之年。政府工作的总体思路：坚定以习近平新时代中国特色社会主义思想为指导，把党的政治建设摆在首位，坚定维护以习近平同志为核心的党中央权威和集中统一领导，全面深入贯彻党的十九大精神和中央、省委省政府决策部署，认真落实省委“一二三四”战略谋划和市委“一个目标、两篇文章、三大工作抓手、四个加快建设”工作思路，始终保持专注发展转型发展战略定力，坚持稳中求进工作总基调，按照高质量发展要求，统筹推进稳增长、促改革、调结构、惠民生、防风险各项工作，打好“三大攻坚战”，加快在全省高水平率先全面建成小康社会步伐，奋力开创美丽繁荣和谐攀枝花建设新局面。

　　2018年全市经济社会发展的主要预期目标：地区生产总值增长7.5%，全社会固定资产投资增长8%，地方一般公共预算收入同口径增长6%，社会消费品零售总额增长10%，城镇、农村居民人均可支配收入分别增长8.5%、9.5%，城镇登记失业率控制在4.2%以内，居民消费价格指数涨幅控制在3.5%以内，节能减排完成省政府下达任务。地区生产总值增长7.5%的目标，与全省基本持平，是积极稳妥的、有支撑的，既体现积极作为，又为转型发展预留了一定空间。

　　重点要做好以下十个方面的工作。

　　一、坚持稳增长调结构，推动经济高质量发展

　　用习近平新时代中国特色社会主义经济思想来审视大势、更新观念、推动发展。坚持党对经济工作的集中统一领导，坚持以人民为中心的发展思想，坚持适应把握引领经济发展新常态，坚持使市场在资源配置中起决定性作用、更好发挥政府作用，坚持适应经济发展主要矛盾变化完善调控政策，坚持问题导向部署经济发展新举措，坚持正确工作策略和方法。

　　做实钒钛文章。全力推进工业强市，完成工业总产值2250亿元，规上工业增加值增长8.5%。加快构建现代产业体系，巩固发展钒钛钢铁等传统优势产业，积极培育钒钛新材料、石墨及石墨烯材料、节能环保装备等战略新兴产业，不断提升产业发展层次和水平。加快工业园区基础设施建设，完成投资12亿元。强化重大产业项目支撑，着力引进一批钒钛深加工项目，新开工26个、新投产30个、续建38个上亿元工业项目。大力发展实体经济尤其是制造业对工业强市至关重要，确保新增钒钛深加工等先进制造业企业10户、规模以上工业企业50户。

　　做好阳光文章。纵深推进5个“康养+”产业，高水平创建中国阳光康养产业发展示范区。做优“康养+农业”，加快推进田园综合体等十大重点项目建设，培育发展观光农业、体验农业等新型业态。做精“康养+工业”，加快建设康复辅助器具产业园，建成康养大数据平台。做实“康养+医疗”，加快建设攀西人类辅助生殖中心、医养康复示范中心等项目，深化与北京阜外医院、四川大学华西医院等的合作。做强“康养+旅游”，加快建设花舞人间、普达等投资亿元以上在建旅游项目，打造东方太阳谷。做活“康养+运动”，举办环攀枝花国际公路自行车赛等高水平赛事，建设攀枝花国家登山健身步道等一批重点项目。

　　强化抓项目、抓政策、抓资金。充分发挥投资对经济增长的关键性作用，深化“项目年”活动，加快推进银江水电站等重大项目建设，完成固定资产投资805亿元以上，交通、污水处理等重大基础设施投资250亿元，工业投资260亿元，技改投资240亿元，服务业投资100亿元，农业投资40亿元。争取各类试点、示范基地、服务平台等政策，深入推进攀西试验区“五大领域”先行先试政策，推动省上支持攀枝花矿业城市转型发展的12条措施落地。规范有序推进政府和社会资本合作，引导民间资本参与重大项目建设。向上争取财政性资金增长8%以上。

　　加快发展现代服务业。充分发挥消费对经济增长的基础性作用，新增限上企业20户以上，服务业增加值增长8.5%。大力培育发展现代物流、研发设计、创业孵化、科技服务等现代服务业。完成房地产投资60亿元、商品房销售150万平方米以上。全面启动全国阳光康养旅游目的地、国家全域旅游示范区、全国旅游休闲示范城市创建工作，大力推进“厕所革命”，旅游总收入突破335亿元、增长20%以上。

　　实施乡村振兴战略。加快建设红格等5个特色小镇、14个“百镇建设”试点镇，完成7个古村落、民俗村落改造，建设40个幸福美丽新村，创建20个省级“四好村”。推进重点水利工程和农田水利基本建设。大力发展现代特色农业，引进实施特色花卉等一批产业重大项目。提升攀枝花芒果、枇杷等区域公用品牌影响力，新培育芒果、蔬菜等出口备案企业6家，农产品出口增长20%以上。促进台湾农民创业园加快发展。启动农产品精深加工园规划建设。农业增加值增长4%。

　　二、构建立体交通体系，加快建设区域性综合交通枢纽

　　重大基础设施首攻交通，举全市之力打好交通建设硬仗，加快补齐通行不畅和物流成本较高的短板，夯实四川南向门户基础，建设综合交通枢纽迈出坚实步伐。全年完成交通基础设施投资46亿元。

　　加强航空保障能力建设。新增航线航班，旅客达到30万人次以上。实施保安营机场喻家坪滑坡地质灾害治理。完成新机场选址咨询和气象观测等工作，力争机场迁建纳入中国民航发展“十三五”规划中期调整。完成通用机场选址咨询报告。

　　加快建设高铁和区域高速路网。加快成昆铁路扩能改造。全力争取攀大（大理）铁路按照不低于250公里/小时的客运专线规划设计、攀昭（昭通）铁路按高铁标准纳入“十三五”规划。编制完成攀盐（凉山盐源）高速公路项目工程可行性研究报告，实质开工建设宜（宜宾）攀高速公路宁南至攀枝花段主体工程。铁路和高速公路完成年度投资26亿元。

　　加快完善市域交通体系。加快炳三区至报社隧道工程等市域交通项目建设进度，完成投资7亿元。启动城市立体交通系统规划建设，解决交通拥堵和停车难、停车乱问题。提升国省干线路网和县域交通水平，建好、管好、护好、运营好农村公路，解决边远乡镇通行难问题。

　　三、推动三大变革，深化供给侧结构性改革

　　坚持以供给侧结构性改革为主线，推动经济发展质量变革、效率变革、动力变革，提高全要素生产率。

　　持续推进“三去一降一补”。把质量变革作为主攻方向，加快淘汰落后产能，完成120户“散乱污”企业清理整治。争取专线供电试点，新增直购电企业16户，争取铁路运费优惠2.8亿元，实现企业电费、运费“两降低”，促进企业研发投入、人才吸引力“两提高”。创建国家级质量强市示范城市。

　　深化国企改革支持民营经济发展。把效率变革作为改革重点，深化国有企业市场化改革，降低负债率，国有企业收益增长10%以上。深入推进混合所有制改革。全面落实促进民间投资的政策措施，推动民营中小微企业梯队成长。

　　深化农业供给侧结构性改革。加快农村土地制度改革，完善农村“三权分置”制度，落实好第二轮土地承包到期后再延长三十年的利好政策。加快农村集体产权制度改革，深化集体林权制度和国有林场、林区改革，稳步推进农业水价综合改革。推动土地托管、土地入股等多种形式的适度规模经营，促进农业发展、农村繁荣、农民增收。

　　积极推动金融改革发展。把动力变革作为重要抓手，完善财政金融互动政策，持续开展重点项目、重点行业、重点企业融资对接，引导金融为重大基础设施建设、城镇化建设和产业发展提供直接资金支持，促进形成金融与实体经济、房地产以及金融系统内部良性循环。加快引进一批证券、银行、保险等金融机构，加大对小微企业金融服务，支持市商业银行走出去。

　　四、推进全面创新改革，以战略资源创新开发引领转型发展

　　扎实抓好全面创新改革“一号工程”，不断增强创新驱动转型发展的辐射带动力，让经济发展更具活力更有韧性。

　　攀西试验区建设取得新进展。持续推进“五个一工程”。加大部省市共建力度，争创国家钒钛材料工程研究中心、国家钒钛技术创新研发中心，做强攀钢钒钛资源综合利用国家重点实验室，组建全国钒钛磁铁矿综合利用标准化技术委员会，建成攀西钒钛检验检测院，国家钒钛交易所取得实质性进展。钒钛高新区工业投资22亿元以上，工业销售收入140亿元以上。

　　实施创新驱动发展战略。促成国家层面对钒钛综合利用的重大关键技术开展科技攻关。完善重大科技攻关协同推进机制，开启与乌克兰钛研究院等的国际合作。引进落地院士工作站2个以上，建成1个“国家千人计划”工作站。促进军民融合发展，加强与中船重工等军工集团的项目合作，争取更多企业获得军工产品资质。创建知识产权强市、国家创新型城市，新增高新技术企业5家，高新技术企业占规上企业比重达12%，高新技术产业产值500亿元以上、占工业总产值比重超过26%，科技研发投入15亿元，占GDP的比重达1.5%左右，专利申请量2600件以上，增长4%。加快科技成果转化运用，科技对经济增长贡献率达到56.5%。力争新增国家级双创载体2家，省级双创载体3家。加大《攀枝花人才新政七条》实施力度。

　　加大重点领域改革力度。纵深推进“9张清单”落地落实。西区国家级基层政务公开标准化规范化试点取得可复制经验。深化商事制度改革，力争全市新登记各类市场主体1.5万户。推动电力改革，年内实现电力市场化交易。落实环保机构垂直改革要求和国家投融资、社会保障、财税改革政策。统筹推进教育、医疗、文化、体育等社会领域改革。

　　五、提升开放合作水平，加快建设四川南向门户

　　全面对接全省“四项重点工程”，深化国际经贸合作，推进开放合作迈向高水平。

　　推进对外开放。加大对重点地区开放力度，主动融入长江经济带、“一带一路”、东盟自由贸易区及中印孟缅经济走廊建设，拓展对外贸易，推动更多钢铁、钒钛、特色农产品出口。

　　深化区域合作。抢抓省委大力推进“四项重点工程”机遇，积极推进与天府新区的产业合作，承接产业转移；提前开展与天府国际机场航班航线对接，促进互联互通；用好自贸区平台，利用“蓉欧班列”加大对外贸易。协调丽江、大理、楚雄等市（州），加快推进丽（丽江）攀、攀大（大理）高速公路云南段建设。深化与北京、上海、成都、苏州、无锡、常州等城市的合作。

　　强化专业化招商。精准搭建合作平台，提高专业化招商能力，围绕钒钛深加工和广泛运用，强化补链强链招商。抓实产业部门招商，围绕“1+5+N”重点产业，利用西博会等活动平台，加大外资项目招引力度，引进实际到位资金700亿元。

　　六、坚决打好“三大攻坚战”，夯实在全省高水平率先全面建成小康社会基础

　　打好防范化解重大风险、精准脱贫、污染防治攻坚战，是关系群众利益的重点，也是我市能否在全省高水平率先全面建成小康社会的关键点。

　　防范化解重大风险。加大金融风险防控，支持银行机构加大不良贷款管理力度，降低银行不良贷款率。防范化解政府债务风险，确保政府债务率下降10%左右。妥善处置房地产领域遗留问题，保持房地产市场平稳健康发展。加强社会稳定风险管控，从源头上预防和减少信访突出问题，实现信访批次、人次“两下降”，信访化解率、群众信访满意率“两上升”。健全立体化社会治安防控体系，深入推进平安攀枝花建设。严格落实安全生产责任制，确保安全生产形势持续稳定向好。提高防灾减灾救灾能力。落实粮食安全行政首长负责制。创建食品药品安全示范城市。

　　全面打赢脱贫攻坚战。高质量推进精准扶贫、精准脱贫，重点开展产业扶贫、就业扶贫、教育扶贫、健康扶贫，积极发展壮大村级集体经济。完善财政投入稳定增长机制，确保财政专项扶贫资金增幅达到35%以上。开展扶贫领域专项整治，严格执行贫困村退出标准，确保1020名贫困人口易地扶贫搬迁、剩余15个贫困村退出、6636名贫困人口脱贫任务全部完成。做好大中型水利水电移民及后扶工作。

　　大力推进大气、水、土壤污染防治。高质量完成中央、省环保督察反馈问题的整改。加强大气污染防治，确保空气质量优良率95%以上。全面落实河长制，加强水源地保护，加快县、乡污水处理设施建设，力争地表水、饮用水水源地水质达标率100%。打好净土保卫战，实施苏铁国家自然保护区矿山迹地治理等重点项目，危险废物处置利用率达到100%。

　　七、加大宜居城市建设力度，加快建设全国阳光康养旅游目的地

　　打好创建全国文明城市硬仗，进一步完善城市功能，优化城市管理，建设宜居城市。

　　提高城市设计和建设水平。加强城市设计管理，完成城市总体设计、“城市双修”规划编制和棚改腾空区、特色小镇等专项设计，逐步降低城区人口密度、建筑密度、建设强度，增强城市空间的层次感和通透性。加快建筑产业现代化和绿色建筑发展，推进装配式建筑产业发展，实现建筑业产值180亿元。加快推进火车南站等运输场站建设，观音岩引水工程实现主城区全面供水。实施主城区主要道路路面改造和城区道路边坡整治。深入开展全国老旧小区改造试点，完成危旧房棚户区改造2.46万户和“三供一业”改造移交。加快新区建设，提升医疗、旅游、康养、家政配套水平。

　　全面启动全国文明城市创建工作。坚持“全面创建、全域创建、全民创建”的总体思路，健全工作机制，实施信仰信念、文明素质、基础设施、环境优化、安全畅通、氛围营造等文明城市创建六大重点工程。扎实推进城乡环境综合整治。加强建成区网格化管理，规范完善小区物业管理服务。加快推进智慧城市建设，完善数字化城管运行体系。

　　八、坚持绿色发展，筑牢长江上游重要生态屏障

　　落实绿水青山就是金山银山的绿色发展理念，不断巩固提升美丽攀枝花的生态环境质量，加快打造绿色转型发展示范城市。

　　推进形成绿色生产生活方式。大力发展节能环保、清洁生产、清洁能源、生态循环农业。倡导简约适度、绿色低碳的生活方式，推进节约型机关、绿色家庭、绿色学校、绿色社区创建。加快建设清洁能源示范市，缅气入攀项目实现主城区通气，生活垃圾焚烧发电厂投产运营。

　　加大生态环境保护修复力度。严守生态保护红线。持续深入开展山水林田湖生态修复，实施重要生态系统保护和工矿废弃地修复重大工程。启动攀枝花湿地公园建设。建立生态环境损害赔偿制度，强化监管执法，坚决制止和惩处生态环境破坏行为。

　　深入实施大规模绿化攀枝花行动。抓好新一轮退耕还林重点工程，大力开展植树造林，加强森林管护。巩固提升国家森林城市、国家园林城市创建成果，加快推进攀枝花公园改造提升和绿道项目建设，让天更蓝、地更绿、水更清。

　　九、坚持以人民为中心的发展思想，提高保障和改善民生水平

　　完善公共服务体系，不断满足人民日益增长的美好生活需要，努力使城市发展更有温度。

　　大力兴办民生实事。在全面完成十大民生工程的基础上，扎实办好公共厕所、公办幼儿园、公共停车场、农贸市场、老旧小区改造、公园绿地、医疗基础设施、中小学设施、交通基础设施等9个方面34项民生实事，提高民生工作精准性、实效性。

　　抓好就业促进和社会保障。确保新增就业1.55万人。建立统一的社会保障公共服务平台，增强养老保险、失业保险兑付保障能力。健全社会救助体系，强化农村留守儿童关爱保护，提升困境儿童保障水平。建立健全“租售补”并举住房保障机制。

　　优质均衡发展社会事业。坚持教育优先发展战略，进一步提高教育质量和水平，提高公办和普惠性幼儿园学位占比，统筹推进城乡义务教育一体化发展，高中阶段教育普及率达到94%，加强职业教育，支持攀枝花学院内涵式发展。推进“三线文化”国家级品牌打造，争创第四批国家公共文化服务体系示范区。实施健康攀枝花战略，加快推进花城新区医院、市妇女儿童医院建设，开展康养医疗基础性科学研究，积极推进家庭医生签约服务和医保付费方式等改革，提高基层医疗机构服务水平、医护人员专业化水平和医疗装备水平。

　　十、提升专业能力、培养专业精神，全面推进法治政府、创新政府、廉洁政府和服务型政府建设

　　深入学习贯彻习近平新时代中国特色社会主义思想和党的十九大精神，全面加强政府系统党的政治建设，坚定维护以习近平同志为核心的党中央权威和集中统一领导，确保党中央大政方针和省委省政府决策部署落地落实。牢固树立“四个意识”，坚定“四个自信”，认真开展“不忘初心、牢记使命”主题教育，深化“两转一提”，努力建设人民满意政府。

　　全面推进依法行政。坚持依法决策，推进合法性审查全覆盖。增强人大意识，自觉接受人大法律监督、工作监督和政协民主监督，坚决执行人大及其常委会的各项决议决定，认真办理人大代表建议和政协委员提案。常态化听取人大代表、政协委员、各民主党派、工商联、知联会、新阶联、无党派人士和人民团体的意见建议，广泛接受群众、媒体和社会监督。加强市场监管、维护市场秩序，强化行政执法监督，规范行政执法行为。加强审计监督。进一步规范招投标活动，加强政府投资建设项目和政府采购过程管理。深化政府信息公开。

　　提升政府服务效能。落实“最多跑一次”要求，深化“放管服”改革，深入推进项目并联、容缺审批。把群众的小事当大事来做，把企业的烦心事当自己的事来办，直接面对群众和企业的政务服务、行政审批还要进一步下沉到县（区）和乡（镇），提高网上办理率，政务服务更加简便高效，进一步优化营商环境。全面实现国际贸易“单一窗口”进出口货物全申报。深入推进专业化招商、项目竞进拉练、项目精细化管理、双向交叉测评，提升工作效能。加强目标督查考核，不断提升政府服务水平。

　　扎实开展“两转一提”。坚定用习近平新时代中国特色社会主义思想武装干部头脑，加强思想政治建设，拓视野、破陈规，大力推动观念转变。大兴调查研究之风，坚持目标导向、问题导向抓落实，整肃庸政懒政怠政行为，健全正向激励和容错纠错机制，推动形成勇于担当、真抓实干的良好政风。加强习近平新时代中国特色社会主义经济思想、产业经济政策法规和相关专业知识的教育培训，全面提升干部队伍的学习本领、政治领导本领、改革创新本领等“八种本领”，确保党的十九大各项决策部署在攀枝花落地落实。

　　实施对标管理。把对标管理作为推动经济社会发展的重要方法，找准我市所处的历史方位和发展坐标，完善对标管理支撑体系、考核机制，既注重与先进城市全面对标，又注重在专业化招商、园区配套、产业发展、科技创新、金融支持、要素保障、行政审批、人才引进等方面单项对标，形成比学赶超、追赶跨越的生动局面。

　　强化党风廉政建设和反腐败工作。严格贯彻落实中央八项规定精神、省市十项规定实施细则，防止“四风”隐形变异、反弹回潮。严格落实“一岗双责”，深化重点领域和岗位的风险防控，持续深入开展党风廉政建设和反腐败斗争。筑牢反腐倡廉思想防线，办好“阳光问廉”，持续正风肃纪，加大查办违纪违法案件力度，举一反三，始终保持惩治腐败高压态势，营造风清气正的干事创业环境。

　　各位代表！使命重在担当，实干铸就辉煌。让我们紧密团结在以习近平同志为核心的党中央周围，高举习近平新时代中国特色社会主义思想伟大旗帜，在省委省政府和市委的坚强领导下，不忘初心、牢记使命，锐意进取、勤勉有为，为建设美丽繁荣和谐攀枝花、开创全市人民美好生活新局面努力奋斗！
